# Chromosome-Level Assembly of *Artemia franciscana* Sheds Light on Sex Chromosome Differentiation

**DOI:** 10.1093/gbe/evae006

**Published:** 2024-01-20

**Authors:** Vincent Kiplangat Bett, Ariana Macon, Beatriz Vicoso, Marwan Elkrewi

**Affiliations:** Institute of Science and Technology Austria (ISTA), Klosterneuburg 3400, Austria; Institute of Science and Technology Austria (ISTA), Klosterneuburg 3400, Austria; Institute of Science and Technology Austria (ISTA), Klosterneuburg 3400, Austria; Institute of Science and Technology Austria (ISTA), Klosterneuburg 3400, Austria

**Keywords:** sex chromosome evolution, genome assembly, dosage compensation

## Abstract

Since the commercialization of brine shrimp (genus *Artemia*) in the 1950s, this lineage, and in particular the model species *Artemia franciscana*, has been the subject of extensive research. However, our understanding of the genetic mechanisms underlying various aspects of their reproductive biology, including sex determination, is still lacking. This is partly due to the scarcity of genomic resources for *Artemia* species and crustaceans in general. Here, we present a chromosome-level genome assembly of *A. franciscana* (Kellogg 1906), from the Great Salt Lake, United States. The genome is 1 GB, and the majority of the genome (81%) is scaffolded into 21 linkage groups using a previously published high-density linkage map. We performed coverage and F_ST_ analyses using male and female genomic and transcriptomic reads to quantify the extent of differentiation between the Z and W chromosomes. Additionally, we quantified the expression levels in male and female heads and gonads and found further evidence for dosage compensation in this species.

**Significance**

Besides its economic importance, the unique characteristics of *Artemia* brine shrimp have made it a great model for exploring many evolutionary questions, including the evolution of sex chromosomes, sexual dimorphism, asexuality, and plasticity of reproductive modes. The genome assembly produced here will be an invaluable resource for advancing the efforts made in elucidating the genetic architecture of evolutionary and biologically relevant traits. It will also pave the way for more comprehensive studies in the phylogenomics and comparative genomics of Arthropods.

## Introduction

*Artemia* brine shrimp are crustaceans belonging to the Anostracan order in the Branchiopoda class, which includes around 1,200 species (Castellucci et al. 2022). They live in saline/hypersaline inland water bodies, with a very wide geographical distribution (Eimanifar et al. 2015). They are very adaptable and can survive in extreme environments; this is facilitated by their ability to produce both live offspring and encapsulated cysts, which can survive in dry conditions for extended periods of time (Criel and Macrae 2002). Their adaptability, ease of rearing, and high nutritional value have made them very popular in the aquarium trade and aquaculture industry (Lavens and Sorgeloos 2000). *Artemia* has other industrial uses, which include controlling algal growth in salt mines and improving the efficiency of salt production (Van Stappen et al. 2020). Furthermore, they have been extensively used in toxicity and ecotoxicity testing due to their abundance, cost-effectiveness, and ease of manipulation in the laboratory (Nunes et al. 2006; Rajabi et al. 2015).

*Artemia franciscana* is arguably the most extensively studied *Artemia* species; however, to date, the genomic resources for *A. franciscana* are limited to 2 scaffold-level assemblies (De Vos et al. 2021; Jo et al. 2021). While they have yielded important insights into the adaptation to extreme environments, several aspects of their reproductive biology, including the molecular basis of sex determination and the extent of sex chromosome differentiation, are difficult to elucidate without a chromosome-level assembly (Huylmans et al. 2019). Currently, the closest relative with a published chromosome-level assembly is the Asian *Artemia sinica* (Elkrewi et al. 2022), from which *A. franciscana* diverged 30 million years ago (Baxevanis et al. 2006; Maniatsi et al. 2009).

*Artemia* are also a great model for sex chromosome evolution, as they have ZW sex chromosomes (Bowen 1963; De Vos et al. 2013; Elkrewi et al. 2022; Boyer et al. 2023). Sex chromosomes are known to evolve from autosomes, when one of them acquires a sex determination gene. Recombination is then thought to be lost in a stepwise manner, creating strata of different ages (Lahn and Page 1999; Handley et al. 2004), but this process is difficult to study in well-differentiated sex chromosomes. An earlier comparison between *A. franciscana* and *A. sinica* suggested that younger strata were acquired independently in the 2 lineages, which would make *Artemia* an ideal model for studying this stepwise process, but the fragmented assembly of *A. franciscana* limited this analysis (Elkrewi et al. 2022). Furthermore, the Artemia genus includes multiple obligate parthenogenetic populations (Abatzopoulos 2018), and the Z chromosome has been implicated in their origin (Elkrewi et al. 2022). In this report, we present a chromosome-level genome assembly for *A. franciscana*, adding a valuable resource to the limited number of anostracan genomes, and use it to characterize the sex chromosome pair at the genomic and gene expression levels.

## Results and Discussion

### A Chromosome-Level Genome Assembly

We generated 5,006,105 PacBio circular consensus reads (CCS). Since an assembly of all the reads did not yield resolved Z and W haplotypes, we used female-specific kmers, generated using a k-mer subtraction approach with female and male short reads (Carvalho and Clark 2013; Elkrewi et al. 2021), to remove CCS reads originating from the W chromosome (25,784 reads, 0.52%; supplementary fig. S1, Supplementary Material online). This was performed to avoid chimeric Z and W assemblies in the regions that retain some homology. The remaining 4,980,321 reads were assembled into 12,122 contigs using Hifiasm (Cheng et al. 2021). After removing 7,335 contigs representing alternative haplotypes, we scaffolded the assembly using evidence from CCS reads, RNA-seq reads, and previously published genomic mate pairs, resulting in 3,477 scaffolds, with an N50 of 590 KB (supplementary fig. S2, Supplementary Material online).

We used a published high-density linkage map (Han et al. 2021) to anchor the scaffolds into 21 linkage groups. To improve the contiguity of the differentiated part of the Z chromosome (Huylmans et al. 2019), we performed coverage analysis to identify the scaffolds originating from the differentiated region of the Z chromosome (supplementary fig. S3, Supplementary Material online) and anchored them using the linkage group 6 (LG6) (Z chromosome in the linkage map) markers. We then added the anchored differentiated region to the rest of the assembly and used the complete linkage map to assign scaffolds to their respective linkage groups (supplementary fig. S4, Supplementary Material online). The resulting assembly was polished (gap filling and correction) using both the filtered CCS reads and male genomic short reads. The putative W reads (removed in the first step) were assembled separately (supplementary fig. S5, Supplementary Material online), resulting in 506 putative W sequences, which were added to the assembly for the downstream analysis.

**Fig. 1.**
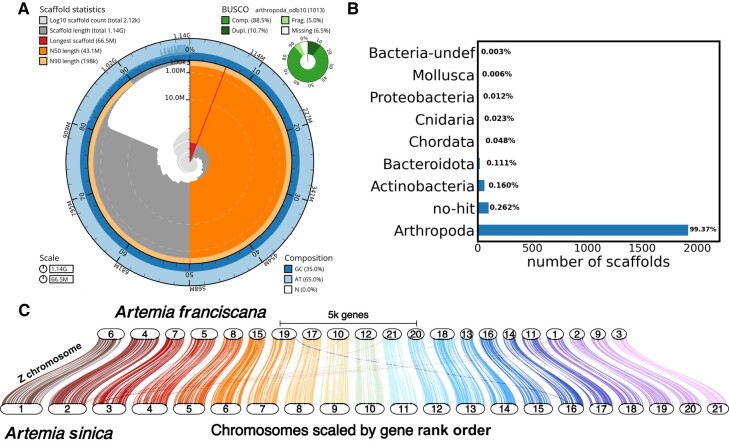
Genome assembly and synteny. a) Genome assembly statistics, GC content, and BUSCO score using the arthropoda_odb10 data set. b) Barplot showing the number of sequences that map to the different phyla in the nt Blast database and the percentage of the total assembly length they represent. c) Synteny between the *A. franciscana* and *A. sinica* genomes.

The final assembly has 2,118 scaffolds and an N50 of 43 MB, with 81% of the assembly in the 21 linkage groups (supplementary table S1, Supplementary Material online). We ran BUSCO to assess the completeness of the genome, and 88.5% of BUSCOs were assembled completely, with <7% missing (Fig. 1a). We also checked for contamination using BlobTools, and the results show that there is <1% bacterial contamination and most sequences map to Arthropoda (Fig. 1b).

As another quality check, we compared our assembly with the *A. sinica* chromosomes. Both the *A. franciscana* and *A. sinica* genomes were annotated using RNA and protein evidence (as described in the Repeat Content Characterization, Genome Annotation, and Synteny between the Artemia Genomes section). The annotations were used to examine and visualize the synteny between the 2 genomes using GENESPACE (Lovell et al. 2022). As Fig. 1c shows, the genomes are highly syntenic, with no evidence for any large-scale rearrangements.

### History and Extent of Differentiation of the Sex Chromosomes

**Fig. 2.**
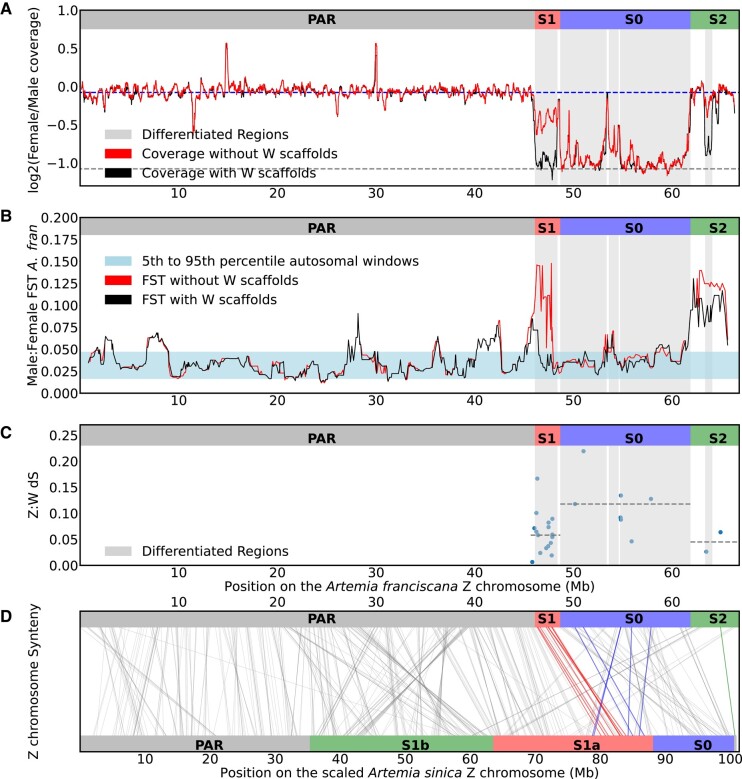
Evolutionary strata of the ZW pair. a) Log2(female/male) coverage patterns of the Z chromosome. The rolling medians of the coverage for 30 consecutive (10,000 bp) windows, estimated with and without including the W scaffolds, are shown in the figure. The vertical shading highlights the differentiated region of the Z chromosomes. The top dashed line is the autosomal median for the analysis without W scaffolds, and the bottom dashed line is at median −1. b) The rolling medians of male:female F_ST_ per gene for 10 genes, estimated with and without including the W scaffolds, are shown. The horizontally shaded area covers the region between the 5th and 95th percentiles for autosomal windows with the W included. c) dS values between the W transcripts and their Z homologs. The dashed lines correspond to the median of the dS values in the different strata. d) Synteny between the *A. sinica* and *A. franciscana* Z chromosomes highlighting the different strata (inferred here for *A. franciscana* and found in Elkrewi et al 2022 for *A. sinica*): S0, S1, and S2. The lines connect the locations of the reciprocal best hits on the chromosomes, with the Z homologs of the identified W transcripts colored according to their strata in *A. franciscana*.

Earlier work suggested the ZW pair of *A. franciscana* has a small but well-differentiated region, which no longer exists on the W, and a nonrecombining but undifferentiated region (Huylmans et al. 2019). We estimated the coverage across the genome using short-read male and female DNA in windows of 10,000 bp (supplementary fig. S6, Supplementary Material online) and then used the ratio of female-to-male coverage to identify regions that have become well differentiated between the Z (LG6) and W chromosomes (Fig. 2a). We performed the analysis once with the W scaffolds included in the assembly and once without them. This makes it possible to identify regions that still share some sequence similarity between the Z and the W, as the W reads originating from those regions will map to the Z chromosome when the W is not included. On the other hand, regions that lost homology completely will have consistent low coverage regardless of whether the W scaffolds are included or not. A 13 MB region of the Z chromosome has coverage in females that is half the male coverage in both analyses. This is in agreement with the results obtained with the *A. franciscana* scaffold-level assembly anchored to the *A. sinica* genome (Elkrewi et al. 2022), but with much greater contiguity of the differentiated region. A smaller region adjacent to it shows a full reduction in female coverage when W scaffolds are included and only partial reduction when they are not, a first line of evidence that it corresponds to a more recent and only moderately differentiated region.

ZW regions with limited differentiation, which show no or only partial coverage differences, can be detected by the presence of genetic variants specific to the W and therefore to females. We used pooled male and female RNA-seq libraries (Huylmans et al. 2019) to estimate the female:male F_ST_, a measure of genetic differentiation, across the genome (supplementary fig. S7, Supplementary Material online). We performed the analysis once with the W scaffolds included in the assembly and once without them. The analysis without the W scaffolds shows elevation in the male:female F_ST_ on the 2 sides of the differentiated region (Fig. 2b). The high F_ST_ is less pronounced on both sides when the W scaffolds are included, as the W-derived RNA reads preferentially map to the W. The decrease is more noticeable on the left side, suggesting a higher degree of differentiation. To further explore this, we estimated the median rate of synonymous substitutions (dS) between the transcripts on the putative W scaffolds which had female-specific expression patterns and their Z homologs for the different regions. We used dS, coverage, and F_ST_ patterns to define 3 strata: the ancestral S0, which shows high dS and consistent low female coverage estimates with and without the W scaffolds; S1, which shows intermediate coverage patterns and elevated F_ST_ when the W scaffolds are not included, suggesting the W reads carry many female-specific variants but still map to the region; and S2, which has elevated F_ST_ in the 2 cases and very low dS. Figure 2a shows the correspondence between those strata and the *A. sinica* strata and where the Z homologs of the identified W transcripts map on the 2 chromosomes. The ZW pairs in *A. franciscana* S0 map to the S1a stratum of *A. sinica*, suggesting that they might not be ancestral.

### Full Dosage Compensation and Repeat Composition on the ZW Pair

**Fig. 3.**
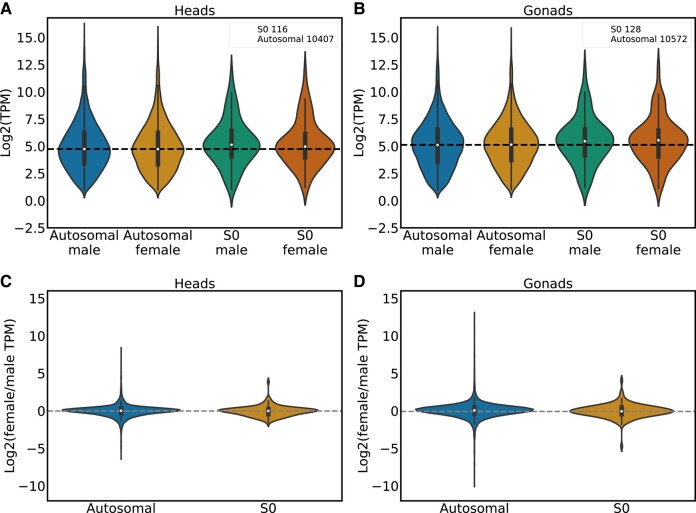
Dosage compensation. a) The Log2 of the expression of autosomal and the Z differentiated region genes in male and female heads. The legend shows the number of genes used in the analysis. b) The Log2 expression of autosomal and the Z differentiated region (S0) genes in gonads. The legend shows the number of genes used in the analysis. c) The Log2 of female/male expression for the autosomal and Z differentiated genes in heads. d) The Log2 of female/male expression for the autosomal and Z differentiated genes in gonads. Only genes with TPM ≥ 0.5 in their respective male and female tissues were used in these analyses.

We estimated levels of expression for all annotated genes from published male and female head and gonad RNA-seq data (Huylmans et al. 2019). Gene expression does not differ between the differentiated region of the Z and the autosomes in either male and female heads or gonads (Fig. 3, *P* > 0.05 with Wilcoxon rank sum tests). This is consistent with dosage compensation, i.e. a mechanism to balance the expression of the sex chromosomes and autosomes in both sexes in species with differentiated sex chromosomes, as reported in earlier work (Huylmans et al. 2019). Most ZW systems that have been studied so far, such as snakes and birds, seem to lack a chromosome-wide mechanism of dosage compensation (Gu and Walters 2017). So far, Lepidoptera (moths and butterflies) have been the only clear exception to this rule (Gu and Walters 2017). Our confirmation that chromosome-wide compensation also occurs in *Artemia* makes it a promising model for understanding why and how such compensatory mechanisms arise in female heterogametic species.

Finally, the absence of recombination between sex chromosomes often leads to the accumulation of transposable elements (TEs) and other repetitive sequences on the Y or W chromosome (Dechaud et al. 2019). Approximately 2/3 of the genome in *A. franciscana* are covered by repetitive elements (supplementary table S2, Supplementary Material online). Retroelements account for 43% of the repeat content in this species. We did not observe striking differences in the overall repeat content between W scaffolds and either S0 region or autosomes. However, W scaffolds that still have homologs to the S0 region, and which should correspond to the most differentiated part of this chromosome, have more retroelements (48%) in relation to those in S0 region, autosomes, and W scaffolds (supplementary table S2, Supplementary Material online). Overall, even this region of the W only shows a modest enrichment in repeats compared with the autosomes (67% vs. 66%) and none relative to the S0 region of the Z (68%). While it is possible that this pattern reflects a slow accumulation of repeats in the nonrecombining part of the W in this clade (or limited power to resolve repeats in the assembly), another possibility is that the ancestral nonrecombining region of the W has been lost entirely and that all regions studied here are relatively young. Complete loss of the original W-specific region would also account for the lack of ZW homologs that map to the S0 of both *A. franciscana* and *A. sinica* and potentially explain why a global mechanism of dosage compensation was selected for in this lineage.

## Materials and Methods

### DNA Extraction and Sequencing

High molecular weight DNA was extracted from 2 unmated females from the great salt lake, purchased from Sanders (Utah, United States), with the Qiagen Genomic-Tip 20/G Kit, and sequenced on a PacBio Sequel II SMRT cell at the Vienna Biocenter sequencing facility. The number of females was chosen to ensure enough genetic material and also limit the amount of genetic variability, which would complicate the assembly.

### Genome Assembly

The consensus sequences of the subreads in the raw bam file were generated using the PacBio ccs tool (option -all) (v6.4.0, https://github.com/PacificBiosciences/ccs). The bbduk.sh script (from BBmap) was used to identify female-specific 21-mers from *A. franciscana* male (SRR19741748) and female short reads (SRR19741747), and the resulting kmers were used to remove putative W-specific CCS reads (reads with 20% or more female-specific kmers, 0.2 mkf) (Bushnell 2014). The filtered CCS reads were then assembled using Hifiasm (--hg-size 1g --n-hap 4 -r 5 -s 0.7 -N 150) (v0.19.4, Cheng et al. 2021). The primary assembly was then purged of duplicates with female short reads using purged_dups (v1.2.5, Guan et al. 2020). We scaffolded the purged assembly with the filtered CCS reads using LongStitch (ntlink + arcs, v1.0.4, Coombe et al. 2021). We independently scaffolded the assembly with male RNA-seq reads (from Huylmans et al. 2019) using Rascaf (Song et al. 2016). We then mapped the 2 resulting assemblies to the input assembly with minimap2 (Li 2018) and used a python script to identify the merges that are supported by both approaches (supplementary fig. S3, Supplementary Material online). RagTag (v2.1.0, Alonge et al. 2022) was used to implement those merges. Redundans (v0.14a, Pryszcz and Gabaldón 2016) was then used with long insert mate pairs (SRR6980924, 8 MB) to further scaffold the assembly.

### Scaffolding Using the Published Linkage Map

The published *A. franciscana* linkage map was used to scaffold the draft assembly into linkage groups (Han et al. 2021). The SLAF markers were mapped to the assembly using BWA-MEM (v0.7.17-r1198-dirty, Li 2013), and the alignments, along with the linkage map, were used by Chromonomer (v1.14, Catchen et al. 2020) to anchor the scaffolds. The scaffolding was done in 2 steps, with the first to anchor the differentiated region of the Z chromosome. To do that, we estimated the coverage for the assembled scaffolds as described in the coverage section and selected putative Z-specific scaffolds (114 scaffolds). We used the perl script BreakScaffolds (https://github.com/aubombarely/GenoToolBox) to split the scaffolds at stretches > 100 Ns, which resulted in 126 scaffolds. The LG6 markers were mapped to those scaffolds, and the LG6 linkage map was used for anchoring (54 scaffolds were anchored into a 13.5 MB region). The output was added to the remaining scaffolds, and the complete linkage map was used to anchor everything into the 21 linkage groups. In order to avoid splitting the differentiated region, we ran 2 iterations of Chromonomer. The first iteration was run with the option “--disable_splitting” to identify the best location for the scaffolded differentiated region. We then modified the original linkage map to retain only the differentiated region markers and location appearing in the output file (CHRR_linkage_map.tsv) from the first iteration. The second iteration was run with the modified linkage map with the option “—rescaffold.” This ensured that Chromonomer was able to break and rescaffold regions with inconsistent markers without splitting the differentiated region.

### Polishing and Quality Assessment

TGS-GapCloser (v1.1.1, Xu et al. 2020) was used to fill the gaps in the assembly using the filtered CCS reads. The first round of polishing with the filtered CCS reads and male short reads used Racon (v1.6.0, Vaser et al. 2017) and Merfin (v1.1, Formenti et al. 2022) through the automated-polishing.sh script (from https://github.com/arangrhie/T2T-Polish/tree/master/automated_polishing, modified from Mc Cartney et al. 2022), and the second round used nextpolish2 (v0.1.0-758ef0a, Hu et al. 2024). The assessment of the assembly completeness was done using BUSCO (v5.2.2), with the arthropoda_odb10 data set (Manni et al. 2021), and BlobTools was used to assess and visualize the level of contamination and genome statistics (Laetsch and Blaxter 2017).

### Genomic Coverage, F_ST_ Analysis, and Identification of the Differentiated Region

For the coverage analysis, male and female Illumina genomic short reads (from Elkrewi et al. 2022) were mapped to the genome using Bowtie2 (--end-to-end --sensitive) (v2.4.4, Langmead and Salzberg 2012). The resulting SAM files were filtered for unique alignments using (grep -vw “XS:i”), and the coverage was estimated for windows of 10,000 bp using soap.coverage (version 2.7.7, https://github.com/gigascience/bgi-soap2/tree/master/tools/soap.coverage/2.7.7).

In the F_ST_ analysis, head and gonad RNA-seq samples from 10 males and 10 females of *A. franciscana* (from Huylmans et al. 2019) were pooled by sex and then mapped to the genome using STAR (v2.7.9a, Dobin et al. 2013). The SAMtools mpileup command was used to generate a text pileup output from the 2 sorted alignment bam files (v1.18, Li et al. 2009). Grenedalf (v0.2.0, Czech et al. 2023) sync was used to get a sync file, which was used as input to the PoPoolation2 perl scripts (create-genewise-sync.pl and fst-sliding.pl) along with the GTF file (Repeat Content Characterization, Genome Annotation, and Synteny between the Artemia Genomes section) to estimate the F_ST_ per gene (Kofler et al. 2011).

The coordinates for the differentiated region (gray-shaded areas; Fig. 2a) were defined using the approach described in Elkrewi et al. (2022), as regions where the Log2(female/male coverage) drops below the median of the Log2(female/male coverage) of autosomal windows (−0.5), and as long as the coverage does not go above the defined threshold, the extension of the region continues. The resulting coordinates were (46,085,001 to 48,385,001), (48,665,001 to 53,365,001), (53,585,001 to 54,575,001), (54,715,001 to 61,855,001), and (63,345,001 to 64,075,001), shaded in gray in Fig. 2a–c.

The annotated W transcripts were mapped to the rest of the transcriptome using BlastN (Altschul et al. 1990), and the reciprocal best hits were identified as the homologs using a customized perl script. W transcripts with homologs on the Z chromosome and the sum of head and gonad female/(male + female) expression ≥ 0.9 were used for estimating the rate of synonymous substitution. The ZW homologs were aligned with the TranslatorX package (Abascal et al. 2010) with the “gblocks” option to filter out unreliable sections of the alignment. The dN and dS values were obtained with KaKs_calculator2.0 (Wang et al. 2010) using the Nei and Gojobori algorithm (NG). Alignments shorter than 300 bp were excluded from the analysis.

### Repeat Content Characterization, Genome Annotation, and Synteny between the *Artemia* Genomes

RepeatModeler (v2.0.4, Flynn et al. 2020) was applied on the *A. franciscana* genome assembly to generate a de novo library of repeat families. The sequences of unknown TEs were classified using DeepTE (Yan et al. 2020) with the options “-sp M -m M.” Subsequently, Class II TEs (DNA transposons) were categorized further into superfamilies and this was done with the following parameters “sp M -m M -fam ClassII.” These were then combined with both Class I known repeat libraries RepeatModeler and DeepTE and used for the characterization of repeat content and masking of the genome by RepeatMasker (v4.1.5, Tarailo-Graovac and Chen 2009). Evidence from both RNA and protein was used for the annotation of predicted genes on the soft-masked genome using BRAKERv2.1.6 (Hoff et al. 2019). For RNA, reads were aligned to the genome using STAR (v2.7.9a, Dobin et al. 2013) in “--twopassMode” and sorted with SAMtools v1.18 (Li et al. 2009). To generate protein hints, all arthropoda protein sequences were downloaded from OrthoDBv11 (https://www.orthodb.org) and then concatenated into a single fasta that was then aligned to the soft-masked genome using ProHint (Brůna et al. 2020) to give predicted protein location in the genome in the form of gff3. BRAKER2, automated gene prediction based on successive runs of GenemarkEP+ and Augustus, was applied on protein hints and sorted RNA alignments with options “–etpmode; –softmasking;.” BRAKER2 was run twice with the second round being performed as before but with an additional hint file generated from the first gene prediction run. AGAT (Dainat et al. 2023) was then used to remove isoforms and incomplete genes without start and/or stop codons and filtered out those with <100 bp length of open reading frames. Protein sequences and coding sequences were extracted using getAnnoFastaFromJoingenes.py, and their completeness was assessed with BUSCO (v5.2.2).

An annotation was produced for the *A. sinica* genome using the same approach described above, and the overall synteny was examined and visualized using GENESPACE v. 0.94 (Lovell et al. 2022). Additionally, the annotated protein sequences for *A. sinica* and *A. franciscana* were mapped to each other using pblat (Wang and Kong 2019) (protein target and query) and reciprocal best hits were found using a customized python script and used for producing Fig. 2d.

### Expression Analysis

Illumina RNA reads of 2 biological replicates of heads and gonads of each sex were mapped to the genome using STAR (v2.7.9a, Dobin et al. 2013) with the following parameters “--twopassMode basic --quantMode TranscriptomeSAM GeneCounts” and additional option --quantTranscriptomeBan IndelSoftclipSingleend to generate bam alignments that are acceptable as inputs to RSEM (Li and Dewey 2011). Transcript abundances of genes (in TPM) were estimated using rsem-calculate-expression in RSEM with options “--paired-end --alignments --estimate-rspd --strandedness reverse”. We then used NormalyzerDE (Willforss et al. 2019) to perform quantile normalization across samples for each tissue separately. We applied different cutoffs of TPM ≥ 0.0, TPM ≥ 0.5, and TPM ≥ 1, in heads and gonads separately, to assess dosage compensation, whereby the genes with average replicate expression above the thresholds were retained in both sexes. The average TPM values for each tissue in each sex were then normalized with Log2 (Fig. 2a–d, supplementary figs. S8a and b and S9a and b, Supplementary Material online).

## Supplementary Material

evae006_Supplementary_Data

## Data Availability

The assembly pipeline and the scripts used in the analysis can be accessed on the gitpage (https://github.com/Melkrewi/Artemia_franciscana_genome/). The raw data are available on the National Center for Biotechnology Information (NCBI) short-read archive (BioProject number PRJNA1017357). The final assembly, the annotation, and the supplementary data sets are available on (https://doi.org/10.15479/AT:ISTA:14705). All genomic and transcriptomic samples used for this study and the steps of the analysis they were used in are described in supplementary table S3, Supplementary Material online.
