# Supplementary material for: Chromosome-Level Assembly of Artemia franciscana Sheds Light on Sex Chromosome Differentiation
Source: Genome Biol Evol. 2024 Jan 20;16(1):evae006. doi: 10.1093/gbe/evae006 (PMC10827361; doi:10.1093/gbe/evae006)
Supplement: evae006_Supplementary_Data [file evae006_supplementary_data.docx]

**Supplementary Material**

**Supplementary Datasets**

Supplementary Dataset 1: *Artemia franciscana* genome assembly and annotation

Supplementary Dataset 2: *Artemia sinica* annotation

Supplementary Dataset 3: Male and Female genomic coverage

Supplementary Dataset 4: Male:female F_ST_ per gene

Supplementary Dataset 5: Normalized gene expression

Supplementary Dataset 6: W-specific sequences

Supplementary Dataset 7: dN/dS results Z-W pairs in *A. franciscana*

All supplementary datasets can be downloaded using the following link: <https://doi.org/10.15479/AT:ISTA:14705>

**Supplementary Figures**


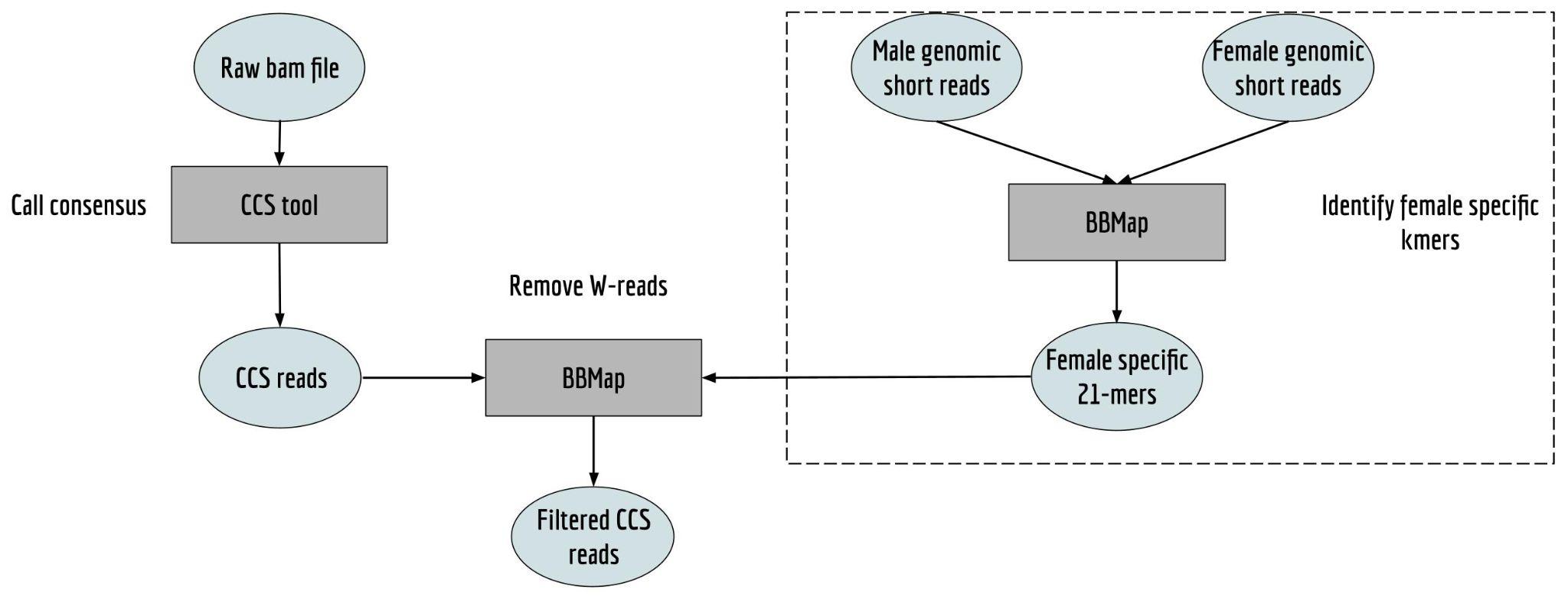


Supplementary Figure 1: the pipeline from generating the consensus reads, and Identifying and removing putative W-reads using female specific k-mers identified using a subtraction approach with male and female genomic short reads.


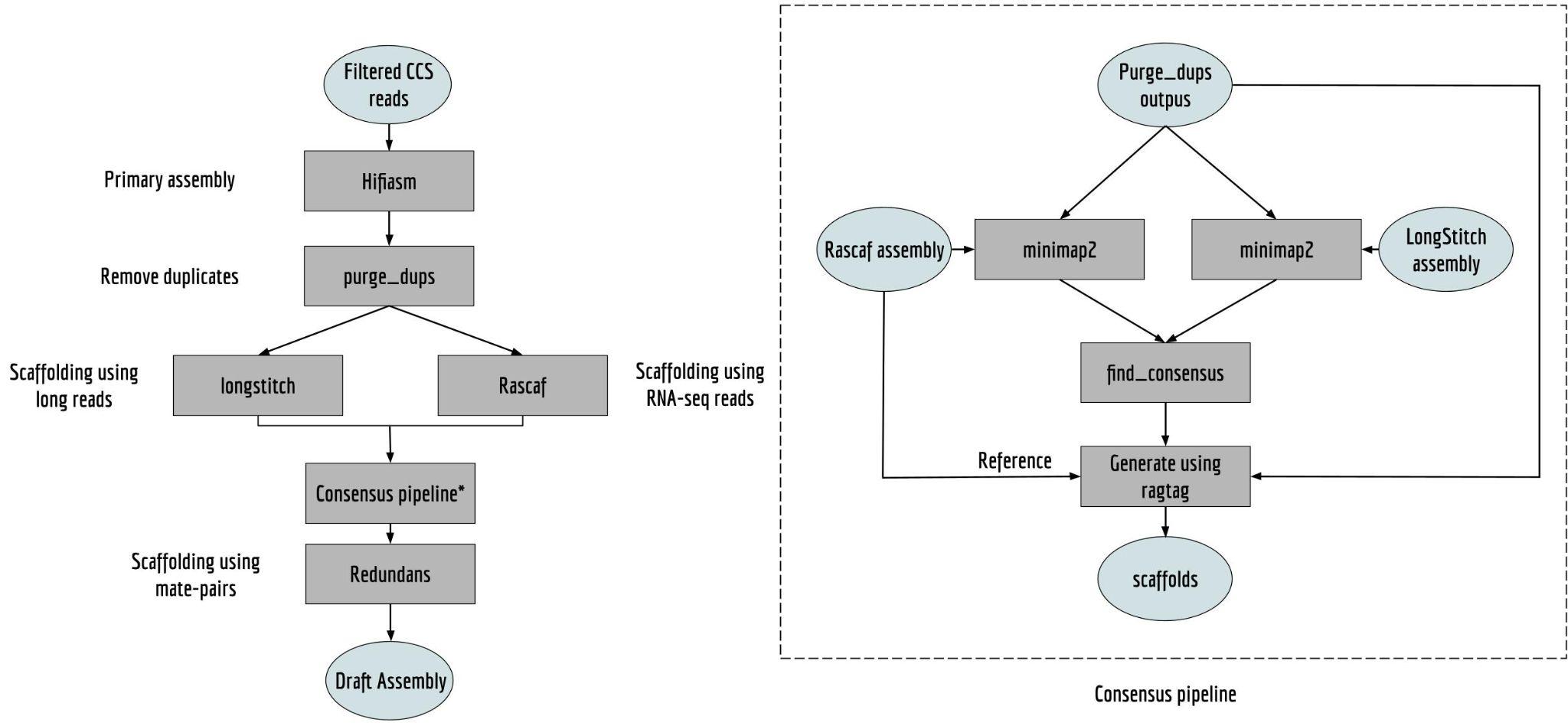


Supplementary Figure 2: Assembling the genome and scaffolding with genomic long reads and RNA-seq reads


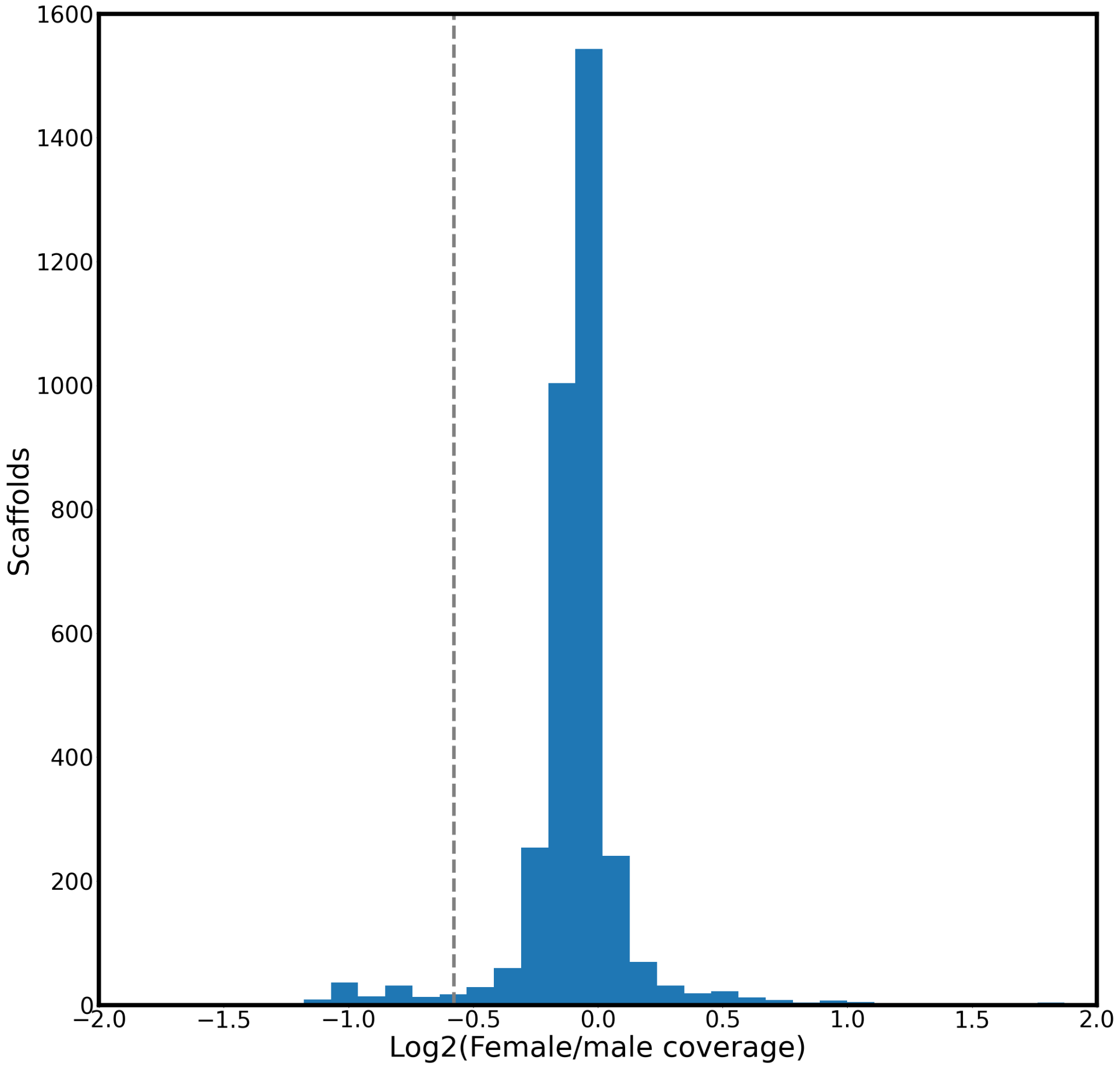


Supplementary Figure 3: Coverage analysis used to identify the scaffolds belonging to the differentiated region of the Z chromosome. The gray-dashed line represents the threshold used: (Log2(female/male) < median( Log2(female/male)-0.5).


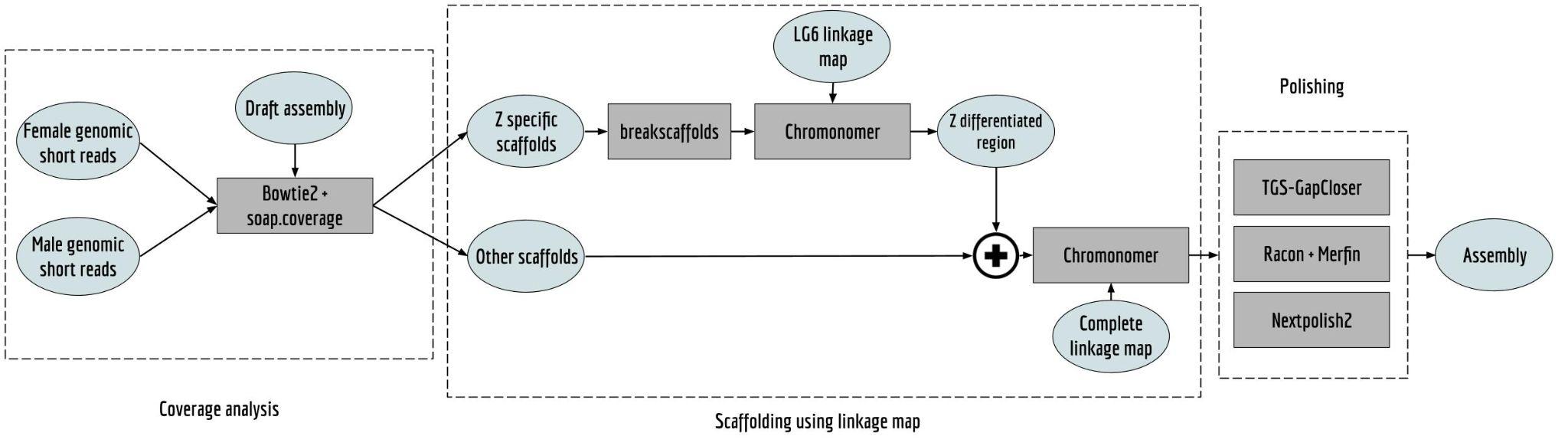


Supplementary Figure 4: The pipeline for scaffolding the genome using the published linkage map

##
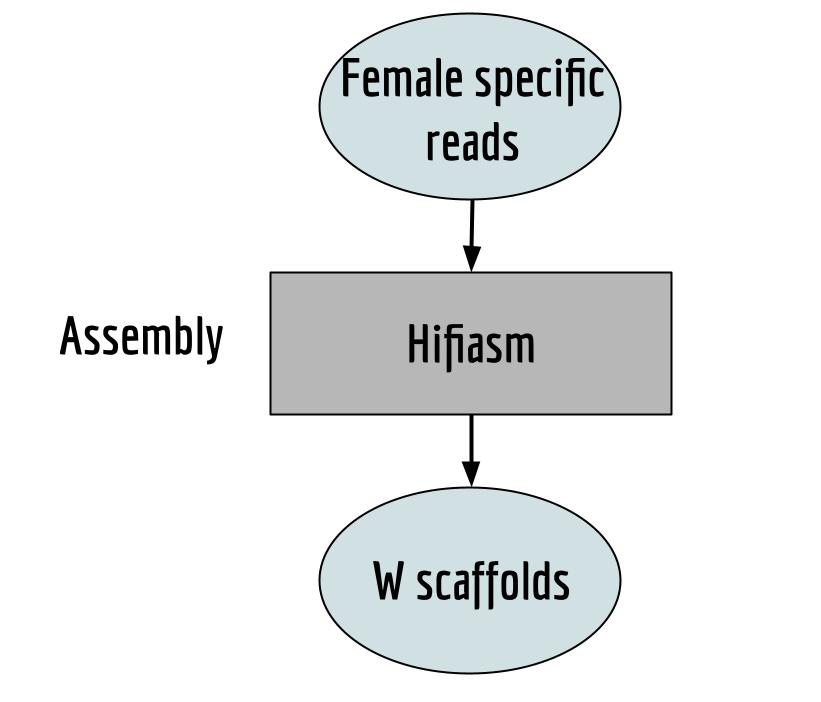


Supplementary Figure 5: Assembly of W scaffolds


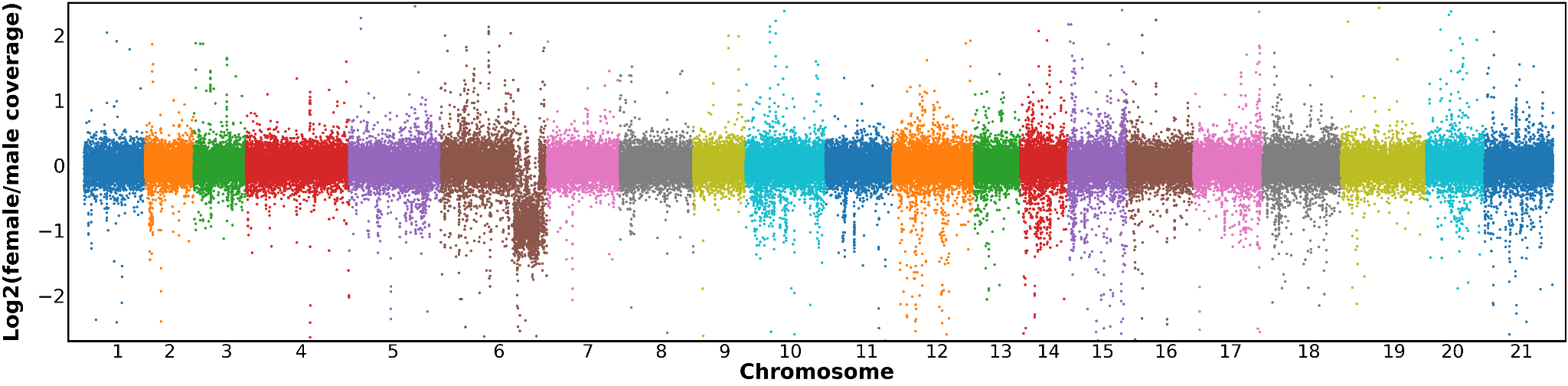


Supplementary Figure 6: Log2(Female/Male) coverage patterns across the whole genome (W-scaffolds included).


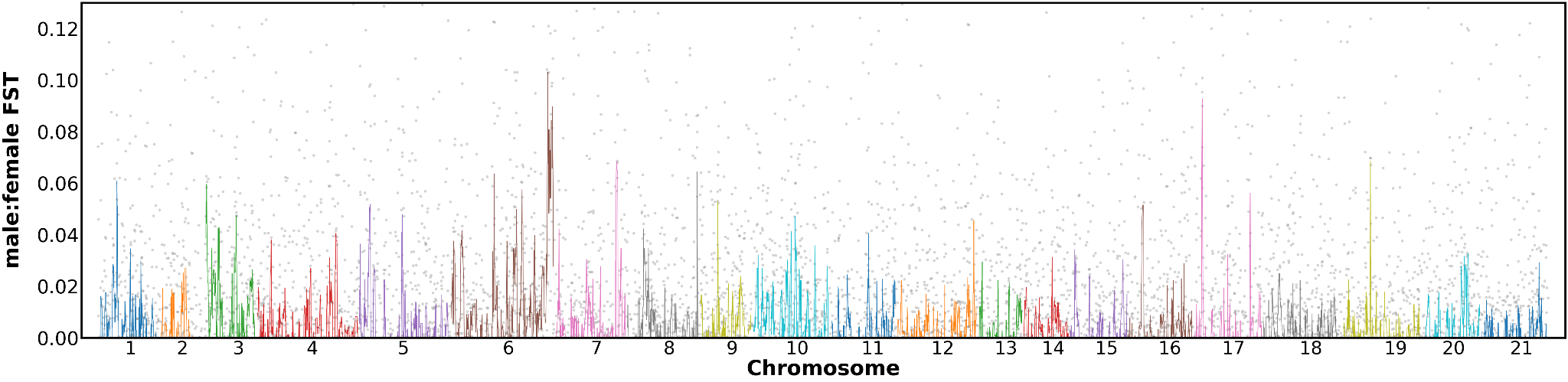


Supplementary Figure 7: male:female F_ST_ patterns across the whole genome (W-scaffolds included). The gray dots are the per gene F_ST_, and the colored tracing is the rolling median of 10 consecutive genes.


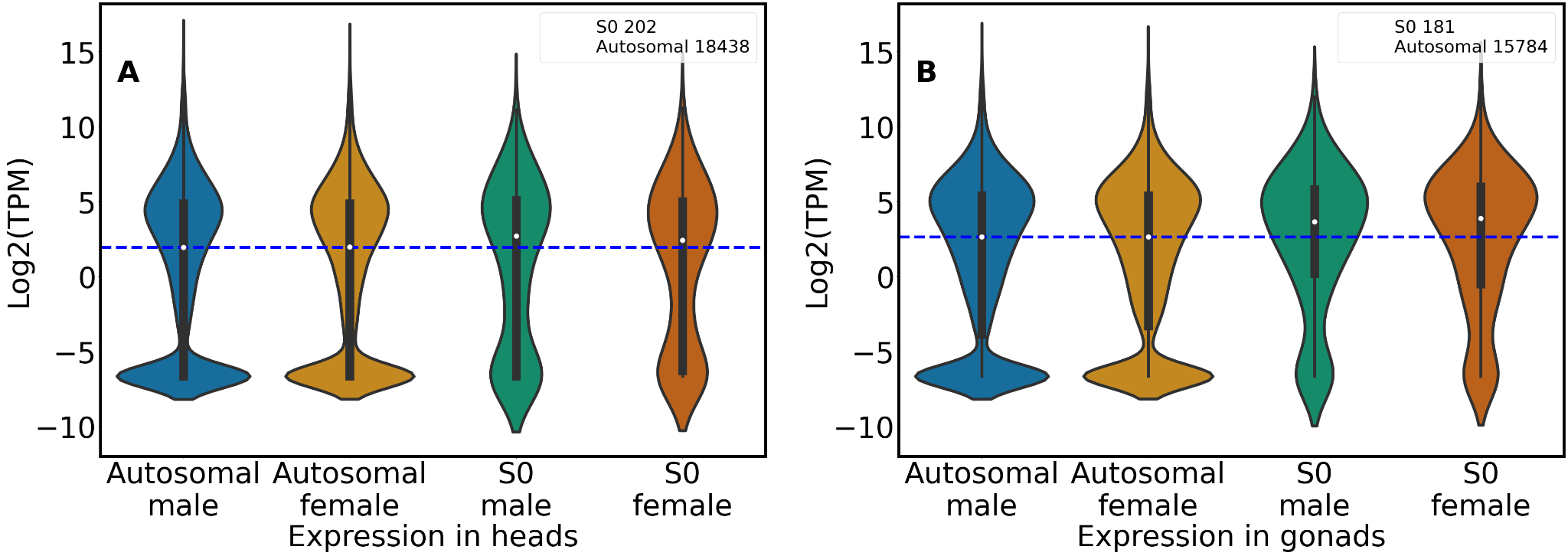


Supplementary Figure 8: A) The Log2 of the expression of Autosomal and the Z differentiated region genes in male and female heads. The legend shows the number of genes used in the analysis (TPM >= 0 in males and females). B) The Log2 expression of Autosomal and the Z differentiated region genes in gonads. The legend shows the number of genes used in the analysis (TPM >= 0 in males and females).


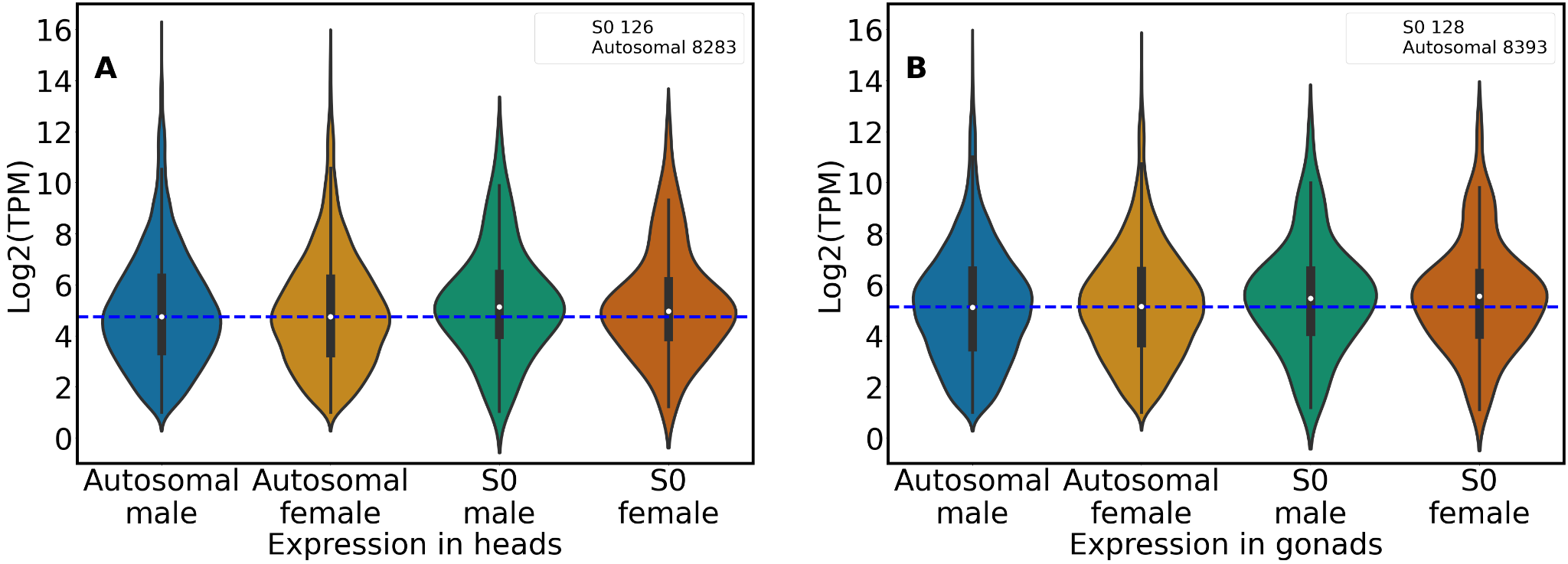


Supplementary Figure 9: A) The Log2 of the expression of Autosomal and the Z differentiated region genes in male and female heads. The legend shows the number of genes used in the analysis (TPM >= 1 in males and females). B) The Log2 expression of Autosomal and the Z differentiated region genes in gonads. The legend shows the number of genes used in the analysis (TPM >= 1 in males and females).

**Supplementary Tables**

Supplementary Table 1: The statistics of the genome and transcriptome assemblies

| Assembly | Genome Size (MB) | 1135.764093 |
| --- | --- | --- |
|  | Number of chromosomes | 21 |
|  | Linkage map anchoring ratio | 81% |
|  | Number of scaffolds/contigs | 2118 |
|  | Longest scaffold/contig (Mb) | 66 |
|  | N50 (MB) | 43 |
|  | Average length (MB) | 0.54 |
|  | Gaps | 2470 |
|  | N_count | 520572 |
|  | GC content (%) | 34.95% |
| Protein-coding genes | Number | 19421 |
|  | Mean gene length (bp) | 30503 |
|  | Exons/introns per gene | ~5.2 exons per gene  ~4.2 introns per gene |
|  | Mean exon/intron length (bp) | ~252 bp exons per gene  ~6964 bp introns per gene |
| Busco of protein sequences of annotated genes | complete | 90.2% |
|  | single-copy | 76.9% |
|  | duplicated | 13.3% |
| Repeats | Size (Mb) | 749 ( 65.94%) |
|  | DNA transposons (MB) | 45 (3.98%) |
|  | SINEs (MB) | 9.2 (0.81%) |
|  | LINEs (MB) | 134 (11.78%) |
|  | LTRs (MB) | 351 (30.91 %) |
|  | Unclassified transposons (Mb) | 125 (11.08%) |

Supplementary Table 2: Proportion of Transposable Elements in W scaffolds, SO region, Autosomes, W-scaffolds that have homologs to S0 region and across whole genome of *A. franciscana*

| **Type** | **Whole genome**  **(bp)** | **Autosomes**  **(bp)** | **ChrZ S0 region**  **(bp)** | **W scaffolds**  **(bp)** | **W scaffolds with S0 homolog**  **(bp)** |
| --- | --- | --- | --- | --- | --- |
| total length | 1135764093 | 1101127100 | 13110000 | 21526993 | 819737 |
| bases masked | 748899995  ( 65.94 %) | 726202062 (65.95%) | 8967129 (68.40%) | 13731055 (63.79 %) | 553123  ( 67.48 %) |
| Retroelements | 494046611 (43.50 %) | 479334586 (43.53 %) | 5500604 (41.96%) | 9211377 (42.79 %) | 396462  (48.36 %) |
| DNA transposons | 5228968  (3.98 %) | 43620029 (3.96 %) | 615807  ( 4.70 %) | 993357 (4.61 %) | 39439  ( 4.81 %) |
| Rolling-circles | 59988424 (5.28 %) | 58336423 (5.30 %) | 1013560  ( 7.73 %) | 638620 (2.97 %) | 15105  (1.84 %) |
| Unclassified | 125827194 (11.08 %) | 121790622 (11.06 %) | 1560033 (11.90 %) | 2475977 (11.50 %) | 89778  (10.95 %) |

## Supplementary Table 3: Genomic and transcriptomic samples used for this study and what steps of the analysis they were used in. This table is available here: <https://github.com/Melkrewi/Artemia_franciscana_genome/blob/048557e7ed5fd57b069516f5c3da652ad9d4c1cf/Artemia_francisca_genome_list_of_samples.xlsx>
